# Effects of dapagliflozin in the progression of atherosclerosis in patients with type 2 diabetes: a meta-analysis of randomized controlled trials

**DOI:** 10.1186/s13098-022-00810-3

**Published:** 2022-03-10

**Authors:** Qian-Long Wu, Ting Zheng, Sheng-Zhen Li, Jin-An Chen, Zi-Chun Xie, Jian-Mei Lai, Ji-Yuan Zeng, Jin-Ting Lin, Jia-Shuan Huang, Min-Hua Lin

**Affiliations:** 1 Guangzhou Medical University, Guangzhou, 511436 China; 2 Department of Rehabilitation Medicine, Hospital for the Aged Guangzhou, Guangzhou, 510550 China

**Keywords:** Dapagliflozin, Atherosclerosis, Type 2 diabetes mellitus, Meta-analysis

## Abstract

**Aims:**

At present, an increasing number of studies are trying to determine whether dapagliflozin has a significant effect on the occurrence and development of atherosclerosis in patients with type 2 diabetes mellitus (T2DM), but there is no consensus. In addition, the former meta-analyses, relying on only a few previous studies and a minimal number of research indicators, have not been able to draw sufficient conclusions simultaneously. Consequently, we conducted a meta-analysis to evaluate the effectiveness of dapagliflozin in the occurrence and development of atherosclerosis in patients with T2DM.

**Methods:**

We searched electronic databases (PubMed, Embase, Cochrane, and Scopus) and reference lists in relevant papers for articles published in 2011–2021. We selected studies that evaluated the effects of dapagliflozin on the risk factors related to the occurrence or development of atherosclerosis in patients with T2DM. A fixed or random-effect model calculated the weighted average difference of dapagliflozin on efficacy, and the factors affecting heterogeneity were determined by Meta-regression analysis.

**Results:**

Twelve randomized controlled trials (18,758 patients) were incorporated in our meta-analysis. In contrast with placebo, dapagliflozin was associated with a significantly increase in high density lipoprotein-cholesterol (HDL-C) [MD = 1.39; 95% CI (0.77, 2.01); *P* < 0.0001], Δflow-mediated vasodilatation (ΔFMD) [MD = 1.22; 95% CI (0.38, 2.06); *P* = 0.005] and estimated Glomerular Filtration Rate(eGFR) [MD = 1.94; 95% CI (1.38, 2.51); *P* < 0.00001]. Furthermore, dapagliflozin had a tremendous advantage in controlling triglycerides (TG) in subgroups whose baseline eGFR < 83 ml/min/1.73m^2^ [MD = − 10.38; 95% CI (− 13.15, − 7.60); *P* < 0.00001], systolic blood pressure (SBP) [MD = − 2.82; 95% CI (− 3.22, − 2.42); *P* < 0.00001], HbA1c, BMI, body weight and waist circumference. However, dapagliflozin has an adverse effect on increasing total cholesterol (TC) and low-density lipoprotein-cholesterol (LDL-C). Besides, there were no significant changes in other indicators, including adiponectin and C-peptide immunoreactivity.

**Conclusions:**

Our pooled analysis suggested that dapagliflozin has a terrifically better influence over HDL-C, ΔFMD, and eGFR, and it concurrently had a tremendous advantage in controlling TG, SBP, DBP, HbA1c, BMI, body weight, and waist circumference, but it also harms increasing TC and LDL-C. Furthermore, this study found that the effect of dapagliflozin that decreases plasma levels of TG is only apparent in subgroups of baseline eGFR < 83 ml/min/1.73m^2^, while the subgroup of baseline eGFR ≥ 83 ml/min/1.73m^2^ does not. Finally, the above results summarize that dapagliflozin could be a therapeutic option for the progression of atherosclerosis in patients with T2DM.

*Systematic review registration* PROSPERO CRD42021278939.

## Introduction

Atherosclerotic cardiovascular disease (ASCVD) is the leading cause of increased morbidity and mortality in patients with diabetes. Statistically, the patients with type 2 diabetes mellitus (T2DM) had approximately twice the risk of cardiovascular death (CVD) events than those without type 2 diabetes, so that cardiovascular protection has become one of the current targets in the treatment of type 2 diabetes mellitus [1–4].

At present, the first-line drug recommended by the clinical guidelines is metformin, which is the primary treatment for T2DM patients [5]. Nevertheless, various glucose-lowering medications differ in mechanism of action and side effects, some of which are also provided with ancillary cardiovascular benefits [6]. Dapagliflozin is a selective sodium-dependent glucose transporter two inhibitors (SGLT2i) that prevents the proximal renal tubules from reabsorbing glucose, thereby inducing glycosuria and lowering glycemia [7]. In addition, Antiatherogenic potential was suggested by animal experiments indicating that SGLT2 inhibition (included dapagliflozin) resulted in increased macrophage-to-feces reverse cholesterol transport, potentially off-loading LDL retention in the arterial intima [8]. At the same time, some clinical trials have also shown that dapagliflozin can decrease ASCVD risk by improving endothelial function or reducing some lipid-related indicators [9]. Abnormalities in lipid are essential in assessing the risk of ASCVD, and optimizing lipid levels remains the primary means to reduce the risk of ASCVD.

Nevertheless, some clinical trials have shown that the overall lipid effects of dapagliflozin did not show clinically meaningful differences among patients with T2DM with and without elevated triglyceride [10]. It means that dapagliflozin has no significant impact on preventing or treating atherosclerosis complications in patients with T2DM. Currently, many studies have attempted to determine whether dapagliflozin has a substantial effect on the progression of atherosclerosis in patients with T2DM, but there is no consensus. In addition, the previous meta-analysis did not get enough published trials and only included a few indicators.

Therefore, we conducted a meta-analysis of clinical studies using dapagliflozin to treat type 2 diabetes over the past decade to explore whether dapagliflozin is an acceptable therapeutic option to inhibit the progression of atherosclerosis.

## Methods

### Information sources

Two investigators independently searched the databases, including PubMed, Embase, Scopus, Cochrane Library and other databases for studies by using the combination of Mesh words and Entry Terms and selected the eligible articles according to the inclusion and exclusion criteria by May 16, 2021. The Mesh words include 'Diabetes Mellitus', 'Sodium-Glucose Transporter 2 Inhibitors', 'Lipid Metabolism', 'Atherosclerosis', 'Endothelium', 'Vascular' and 'clinical trials'. The integrated search formula that we used for PubMed was as follows: (((("dapagliflozin" [Supplementary Concept]) OR ((((((((2S,3R,4R,5S,6R)-2-(4-chloro-3-(4-ethoxybenzyl)phenyl)-6-(hydroxymethyl)tetrahydro-2H-pyran-3,4,5-triol[Title/Abstract]) OR (Farxiga[Title/Abstract])) OR (Forxiga[Title/Abstract])) OR (2-(3-(4-ethoxybenzyl)-4-chlorophenyl)-6-hydroxymethyltetrahydro-2H-pyran-3,4,5-triol[Title/Abstract])) OR (BMS 512148[Title/Abstract])) OR (BMS512148[Title/Abstract])) OR (BMS-512148[Title/Abstract]))) OR (("Sodium-Glucose Transporter 2 Inhibitors"[Mesh]) OR (((((((((((((Sodium Glucose Transporter 2 Inhibitors[Title/Abstract]) OR (Sodium-Glucose Transporter 2 Inhibitor[Title/Abstract])) OR (Sodium Glucose Transporter 2 Inhibitor[Title/Abstract])) OR (SGLT-2 Inhibitors[Title/Abstract])) OR (SGLT 2 Inhibitors[Title/Abstract])) OR (Gliflozins[Title/Abstract])) OR (SGLT2 Inhibitors[Title/Abstract])) OR (Gliflozin[Title/Abstract])) OR (SGLT-2 Inhibitor[Title/Abstract])) OR (Inhibitor, SGLT-2[Title/Abstract])) OR (SGLT 2 Inhibitor[Title/Abstract])) OR (SGLT2 Inhibitor[Title/Abstract])) OR (Inhibitor, SGLT2[Title/Abstract])))) AND (((("Atherosclerosis"[Mesh]) OR ((Atheroscleroses[Title/Abstract]) OR (Atherogenesis[Title/Abstract]))) OR (("Lipid Metabolism"[Mesh]) OR (Metabolism, Lipid[Title/Abstract]))) OR (("Endothelium, Vascular"[Mesh]) OR (((((((Vascular Endothelium[Title/Abstract]) OR (Endotheliums, Vascular[Title/Abstract])) OR (Vascular Endotheliums[Title/Abstract])) OR (Capillary Endothelium[Title/Abstract])) OR (Capillary Endotheliums[Title/Abstract])) OR (Endothelium, Capillary[Title/Abstract])) OR (Endotheliums, Capillary[Title/Abstract]))))) AND (("Diabetes Mellitus"[Mesh]) OR (("Diabetes Mellitus, Type 2"[Mesh]) OR (((((((((((((((((((((((((((((((Diabetes Mellitus, Noninsulin-Dependent[Title/Abstract]) OR (Diabetes Mellitus, Ketosis-Resistant[Title/Abstract])) OR (Diabetes Mellitus, Ketosis Resistant[Title/Abstract])) OR (Ketosis-Resistant Diabetes Mellitus[Title/Abstract])) OR (Diabetes Mellitus, Non-Insulin Dependent[Title/Abstract])) OR (Diabetes Mellitus, Non-Insulin-Dependent[Title/Abstract])) OR (Non-Insulin-Dependent Diabetes Mellitus[Title/Abstract])) OR (Diabetes Mellitus, Stable[Title/Abstract])) OR (Stable Diabetes Mellitus[Title/Abstract])) OR (Diabetes Mellitus, Type II[Title/Abstract])) OR (NIDDM[Title/Abstract])) OR (Diabetes Mellitus, Noninsulin Dependent[Title/Abstract])) OR (Diabetes Mellitus, Maturity-Onset[Title/Abstract])) OR (Diabetes Mellitus, Maturity Onset[Title/Abstract])) OR (Maturity-Onset Diabetes Mellitus[Title/Abstract])) OR (Maturity Onset Diabetes Mellitus[Title/Abstract])) OR (MODY[Title/Abstract])) OR (Diabetes Mellitus, Slow-Onset[Title/Abstract])) OR (Diabetes Mellitus, Slow Onset[Title/Abstract])) OR (Slow-Onset Diabetes Mellitus[Title/Abstract])) OR (Type 2 Diabetes Mellitus[Title/Abstract])) OR (Noninsulin-Dependent Diabetes Mellitus[Title/Abstract])) OR (Noninsulin Dependent Diabetes Mellitus[Title/Abstract])) OR (Maturity-Onset Diabetes[Title/Abstract])) OR (Diabetes, Maturity-Onset[Title/Abstract])) OR (Maturity Onset Diabetes[Title/Abstract])) OR (Type 2 Diabetes[Title/Abstract])) OR (Diabetes, Type 2[Title/Abstract])) OR (Diabetes Mellitus, Adult-Onset[Title/Abstract])) OR (Adult-Onset Diabetes Mellitus[Title/Abstract])) OR (Diabetes Mellitus, Adult Onset[Title/Abstract])))) AND ((((clinical[Title/Abstract] AND trial[Title/Abstract]) OR clinical trials as topic[MeSH Terms] OR clinical trial[Publication Type] OR random*[Title/Abstract] OR random allocation[MeSH Terms] OR therapeutic use[MeSH Subheading]))).

### Inclusion and exclusion criteria

The inclusion criteria were as below:

1. Research type: Randomized controlled trials (RCTs) conducted in any country,
2. An object of study: Patients with T2DM who had or were at high cardiovascular risk, defined as stable coronary artery disease or subclinical carotid atherosclerotic disease,
3. Intervening measure: dapagliflozin intervention or placebo control, and could be combined with metformin or other standards of care therapy for T2DM,
4. Outcome indicator: The study included at least one of the following, including the risk factors related to atherosclerosis, for instance, lipid profile or lipoprotein particles, hemodynamic parameter or endothelial function, BMI, metabolites associated with atherosclerosis, and so forth,
5. The sample size of randomized controlled trials is at least 28,
6. Written in English only.

The exclusion criteria were as below:

1. Non-randomized controlled trials (NRCTs),
2. Randomized controlled studies without blinding, lack of controlled and single case studies,
3. repetitive literature, individual case reports, review articles, empirical perspectives, conference abstracts, and studies without available data,
4. Diabetes with other diseases, such as type 1 diabetes, acute illness or infection, impaired liver or severe renal disease,
5. The efficacy of the risk factors related to atherosclerosis is unable to determine from the trials.

### Data collection

Two reviewers worked on the data collection process on their own. The necessary data and information were extracted and organized into tables by using Microsoft Excel. The following data and information were extracted:

1. Lipid parameters: total cholesterol (TC), low-density lipoprotein-cholesterol (LDL-C), high-density lipoprotein-cholesterol (HDL-C), triglycerides (TG), adiponectin, free fatty acids,
2. Hemodynamic parameter and endothelial function: systolic blood pressure(SBP), diastolic blood pressure(DBP), Δflow-mediated vasodilatation(ΔFMD),
3. Glycemic parameter: HbA1c, fasting plasma glucose (FPG), the number of patients whose HbA1c reached < 7.0%,
4. Metabolic Parameter: BMI, body weight, waist circumference, C-peptide immunoreactivity, estimated Glomerular Filtration Rate(eGFR), urine albumin creatine ratio (UACR),
5. Other variables: participant, gender, country, age, duration of diabetes, rate of dyslipidemia, rate of current tobacco use, diagnosis, intervention characteristics, sources of funding, NCT No.

If there were any discrepancies in the data or disagreements between the two investigators, the third-party investigator would check the study and decide the general policy after proper discussion.

### Missing data

If any necessary data were missing, we contacted the corresponding authors or sponsors for the missing data or information via email. When the average change in the continuous data was not reported with a standard deviation (SD), but 95% confidential interval (95% CI) or P-value of both sets were reported simultaneously, these data were included in the meta-analysis by conversion. When binary data were not available, a sensitivity analysis was conducted for evaluation [11].

### Quality assessment

The quality of the inclusive articles was evaluated by two researchers respectively in conformity with the Cochrane risk of bias tool and Jadad scale. The Cochrane risk of bias tool was applied to assess the risk, including selection bias, performance bias, detection bias, attrition bias, and reporting bias as high, unclear, or low. Review Manager V.5.4 was used to appraise the risk of bias. Furthermore, we used the Jadad scale to appraise the methodological quality of each trail in line with the description of randomization, blinding, and dropouts, accounting for 2 points, 2 points, and 1 point, respectively. Finally, studies that scored 3 points or more were considered high-quality trials. Any divergences generated from this process were passed on to the third investigator, obtaining a consensus.

### Data synthesis

The data of designated studies were analyzed by Review Manager V.5.4. We computed the mean difference (MD) with a 95% CI and obtained the means and SD from continuous results, and random-effects models were used to measure these results. The I^2^ value revealed the heterogeneity of all statistical tests in the forest plots, which was accounted for as follows: 0–40%: exhibiting low heterogeneity; 50–70%: indicating moderate heterogeneity, and > 70%: showing significant heterogeneity [12]. Galbraith's diagram reveals that the heterogeneity created by the circle beyond the lower and upper lines. To comprehend the effect on the results, sensitivity analysis was performed by excluding one literature to evaluate the effect on the result. STATA V.14.0 was applied to subsequent operations. Visual funnel plots and Egger’s test were used to assess the probability of publication bias, and the two-tailed P-value < 0.05 was indicated statistically significant. Moreover, this meta-analysis was conducted based on the Preferred Reporting Items for Systematic Reviews and Meta-Analyses PRISMA guidelines.

## Results

### Study selection

**Fig. 1:**
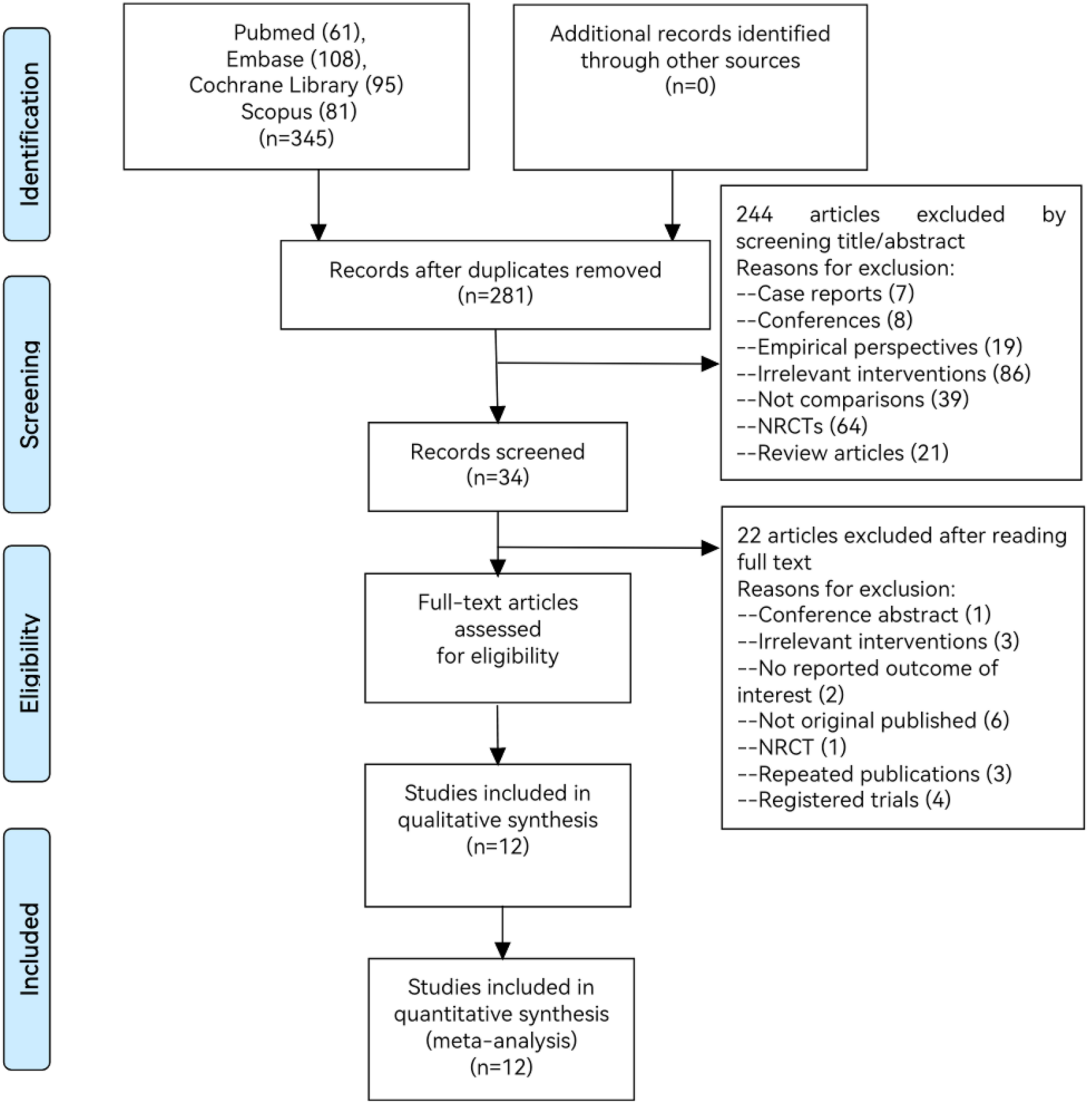
The flowchart of the selection process. In total, twelve studies articles met the eligibility criteria and were included in the meta-analysis

Totally, 345 studies were selected from PubMed (n = 61), Embase (n = 108), Cochrane Library (n = 95), Scopus (n = 81) and the other resources (n = 0). Two hundred and eighty-one references remained after removing duplicate allusions. Through the primary inspecting of titles and abstracts, we excluded 244 articles, including case reports (n = 7), conferences (n = 8), empirical perspectives (n = 19), irrelevant interventions (n = 86), lack of comparisons (n = 39), NRCTs (n = 64) and review articles (n = 21). After remained 34 articles, we supplementary removed 22 articles, consisting of conference abstract (n = 1), irrelevant interventions (n = 3), not originally published (n = 6), NRCT (n = 1), repeated publications (n = 3), registered trials (n = 4), sample size less than 28 (n = 2) and two that had no reported outcome of interest. Finally, twelve references were retained. Methods for each study have previously been published [9, 10, 13–22]. The specific selecting process flowchart is shown in the following Fig. 1.

### Study characteristics

In the twelve clinical trials of 12 to 201.6 weeks' time range (mean = 41.97 weeks, median = 24 weeks), 18,759 diabetes patients were assigned to the dapagliflozin group (n = 9331) or placebo group (n = 9428) randomly. The trial population all accepted 10 mg dapagliflozin or placebo, combined with metformin or other standards of care therapy for T2DM. The mean age varied from 55.2 years to 66.3 years and the gender (male/female) ratio ranges from 2/8 to 28/8. Moreover, the mean duration of diabetes ranged from 5.4 years to 14.2 years, and the mean BMI varied from 26.5 kg/m^2^ to 33.1 kg/m^2^. Furthermore, the mean SBP ranged from 120.0 mmHg to 149.8 mmHg, and the mean DBP varied from 76.3 mmHg to 91.1 mmHg. Not only that, the mean HbA1c in the seven trials ranged from 5.9% to 9.66%, and the mean eGFR ranged from 73.2 mL/min/1.73m^2^ to 101.33 mL/min/1.73m^2^. The main characteristic of each study is summarized in the following Table 1.

**Table 1:** Characteristics of included studies

Trials	Year	Country	NCT No	Patients	Time range (weeks)	Sample size			Mean age (Years)		Gender (Male/female)		Duration of diabetes (Years)	
						Total	Dapagliflozin	Placebo	Dapagliflozin	Placebo	Dapagliflozin	Placebo	Dapagliflozin	Placebo
Bays	2017	North America-U.S.A	NA	T2D^*#^	24	4401	2237	2164	59 (± 15.4)	60.1 (± 15.9)	1280/957	1254/910	9.1	9
Cahn	2021	Europe-Polycentric	NCT01730534	T2D^*#^	201.6	10,186	5108	5078	64.8 (± 5.7)	64.8 (± 5.6)	2874/2234	2839/2239	11.8	11.9
Fadini	2017	Europe-Italy	NCT02327039	T2D^*#^	12	31	15	16	66.3 (± 1.8)	61 (± 1.8)	10/5	11/5	14.2	13.9
Faerch	2021	Oceania-Australia	NCT02695810	T2D^#^	26	60	30	30	61.4 (± 8.5)	57.2 (± 9.9)	13/17	12/18	–-	–-
Hardy	2013	North America-U.S.A	NA	T2D^#^	24	2586	1193	1393	–	–	–	–	–-	–-
Jiang	2021	Asia-China	CTR20150102	T2D^#^	24	29	19	10	58.3 (± 8.01)	59.3 (± 9.03)	9/11	2/8	7.9	7.8
Leiter	2016	North America-Canada	NCT01031680, NCT01042977	T2D^*^	104	568	284	284	63.0 (± 7.3)	62.8 (± 7.4)	197/87	208/76	12.7	11.8
Nur Aisyah	2020	Asia-Malaysia	NA	T2D^#^	12	72	36	36	57.25 (± 8.49)	58.0 (± 7.32)	28/8	27/9	8.94	10.72
Papadopoulou	2021	Europe-Greece	NCT02887677	T2D^#^	12	85	43	42	61.74 (± 6.73)	60.64 (± 9.35)	23/20	21/21	–-	–-
Shigiyama	2017	Asia-Japan	UMIN000018754	T2D^#^	16	74	37	37	57.9 (± 8.3)	59.4 (± 10.1)	25/12	22/15	5.4	6.3
Sugiyama	2018	Asia-Japan	UMIN0000333354	T2D^#^	24	54	27	27	55.2 (± 8.7)	56.0 (± 8.0)	19/8	20/7	10.0	11.0
Weber	2016	North America-U.S.A	NCT01137474	T2D^#^	12	613	302	311	55.6 (± 8.4)	56.2 (± 8.9)	179/123	171/140	8.2	7.6

Trials	BMI (Kg/m2)		SBP(mmHg)		DBP(mmHg)		HbA1c(%)		eGFR(mL/min/1.73m^2^)	
	Dapagliflozin	Placebo	Dapagliflozin	Placebo	Dapagliflozin	Placebo	Dapagliflozin	Placebo	Dapagliflozin	Placebo
Bays	32.3 (± 5.6)	32.2 (± 5.7)	131.7 (± 15.3)	131.8 (± 14.9)	–	–	8.2 (± 0.9)	8.2 (± 0.9)	81.6 (± 19.1)	81.2 (± 19.1)
Cahn	32 (± 5.9)	32 (± 6.1)	135.7(± 15.0)	135.5(± 15.2)	78.4(± 8.9)	78.4(± 8.8)	8.3(± 1.2)	8.3(± 1.2)	85.4(± 15.2)	85.8(± 14.8)
Fadini	28.4(± 1.4)	32.8(± 1.4)	–	–	–	–	8.2(± 0.2)	8.2(± 0.2)	89.3(± 4.4)	92.5(± 4.4)
Faerch	30.9(± 4.5)	32.6(± 6.7)	135(± 14.0)	135 (± 17)	85 (± 7.0)	87 (± 8.0)	5.9 (± 0.19)	5.9 (± 0.26)	84 (± 8.0)	85 (± 7.0)
Hardy	32.4 (± 4.4)	32.4 (± 4.3)	–	–	–	–	–	–	82.1 (± 13.7)	81.9 (± 13.7)
Jiang	26.6 (± 1.32)	25.64 (± 1.41)	123.2 (± 20.27)	120 (± 4.25)	78.16 (± 7.97)	80.2 (± 9.31)	8.1 (± 0.3)	8.5 (± 0.22)	77.2 (± 13.3)	76.1 (± 13.0)
Leiter	33.1 (± 5.3)	32.3 (± 5.6)	133.9 (± 13.9)	133.7 (± 13.2)	78 (± 8.3)	77.2 (± 8.6)	8.1 (± 0.8)	8.1 (± 0.8)	79.74 (−)	79.74 (-)
Nur Aisyah	27.49 (± 4.1)	29.85 (± 4.23)	140.92 (± 22.07)	141.06 (± 21.61)	78.31 (± 11.17)	77.86 (± 12.78)	9.66 (± 1.86)	9.31 (± 1.58)	85.24 (± 18.69)	82.31 (± 15.66)
Papadopoulou	31.33 (± 4.5)	31.83 (± 7.08)	129 (± 12.6)	129 (± 12.4)	77.3 (± 7.3)	78.9 (± 8.7)	7.75 (± 0.57)	7.75 (± 0.42)	101.33 (± 20.85)	95.02 (± 20.05)
Shigiyama	26.8 (± 4.6)	26.3 (± 3.5)	129.2 (± 13.7)	130 (± 12.4)	81.8 (± 9.6)	79.9 (± 8.5)	6.8 (± 0.5)	6.9 (± 0.5)	84.1 (± 19.5)	83.4 (± 17.2)
Sugiyama	27.6 (± 2.2)	26.5 (± 3.1)	130.3 (± 15.3)	131 (± 13.4)	78 (± 8.3)	76.3 (± 7.5)	7.9 (± 0.4)	8 (± 0.3)	75.4 (± 15.4)	73.2 (± 11.8)
Weber	30.21 (± 4.3)	29.52 (± 3.3)	149.8 (± 7.5)	149.5 (± 8.0)	91.1 (± 4.8)	90.8 (± 4.9)	8.1 (± 1.0)	8 (± 0.9)	85.1 (± 18.9)	86.7 (± 19.4)

*NCT No.* National clinical trial number, *T2DM* Type 2 diabetes mellitus

Values are mean (± SD); (–) devotes unclear

* means patients included in the study defined by stable coronary artery disease or subclinical carotid atherosclerotic disease

# means patients included in the study had or were at high cardiovascular risk

### Quality assessment

**Fig. 2:**
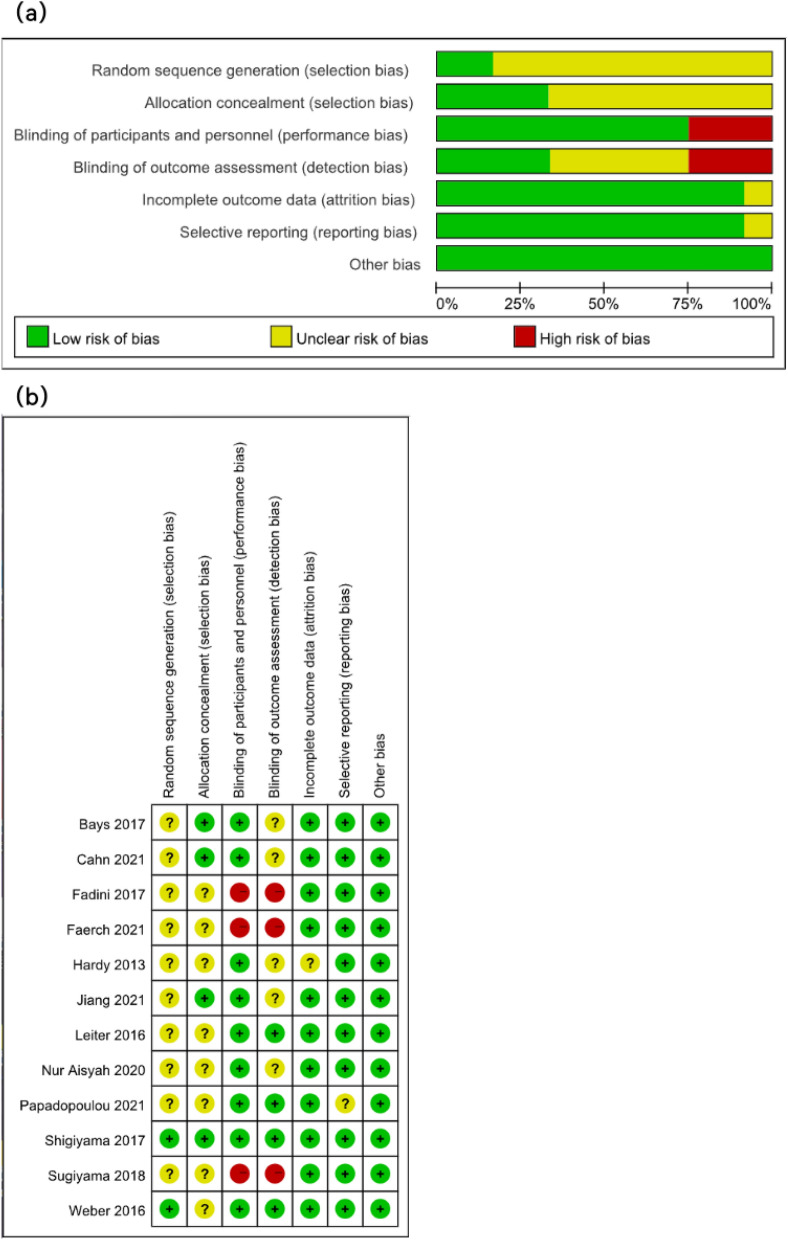
The assessment of the quality of clinical trials is shown. **a** Risk of bias graph. **b** Risk of bias summary. (+) denotes a low risk of bias; (−) denotes a high risk of bias; (?) denotes an unclear risk of bias

The Cochrane risk of bias tool showed that eight articles were evaluated as low risk of bias while one was unclear in two types of selection bias. In the risk of performance bias, nine trials were regarded with low risk, and three articles were concerned with high risk. In terms of the detection bias, four trials were concerned as low risk, and three articles were concerned with high risk; others were unclear. Eleven articles were estimated as low risk in the attrition bias, and only one study was at unclear risk. Reporting bias was at low risk in all trials. The risk of other bias was appraised as low risk (Fig. 2).

Each item of the Jadad scale is scored between 1 and 5, and trials scored three or more were considered high-quality trials. In our meta-analysis, seven trials were scoring 3 points or more assessed as high quality, and five studies were 2 points as low quality (Table 2).

**Table 2:** Jadad Scoring for quality assessment of included trials

Trials	Randomisation mentioned	Concealment of randomisation	Blinding	Appropriate blinding method	Reporting of withdrawals	Jadad score
Bays (2017)	Unclear	Yes	Yes	Unclear	Yes	3
Cahn (2021)	Unclear	Yes	Yes	Unclear	Yes	3
Fadini (2017)	Unclear	Yes	No	No	Yes	3
Faerch (2021)	Unclear	Unclear	No	No	Yes	1
Hardy (2013)	Unclear	Unclear	Yes	Unclear	No	1
Jiang (2021)	Unclear	Unclear	Yes	Unclear	Yes	2
Leiter (2016)	Unclear	Unclear	Yes	Yes	Yes	3
Nur Aisyah (2020)	Unclear	Unclear	Yes	Unclear	Yes	2
Papadopoulou (2021)	Unclear	Unclear	Yes	Yes	Yes	3
Shigiyama (2017)	Yes	Yes	Yes	Yes	Yes	5
Sugiyama (2018)	Unclear	Unclear	No	No	Yes	1
Weber (2016)	Yes	Unclear	Yes	Yes	Yes	3

### Primary outcome

#### Lipid parameters

##### Meta-analysis of the TC changes relative to baseline

**Fig. 3:**
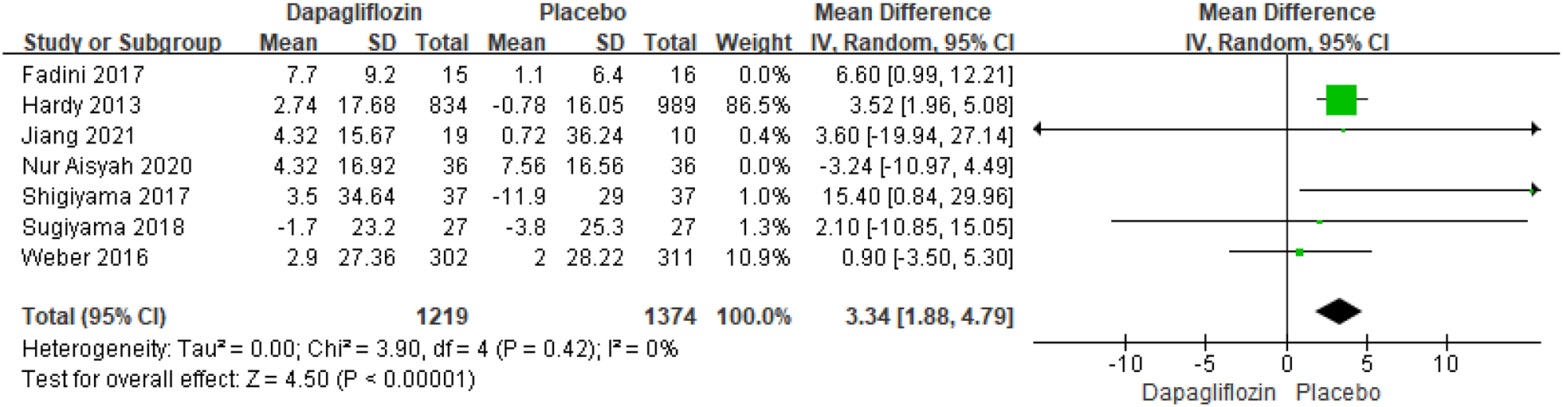
Impact of dapagliflozin on TC changes relative to baseline after deleting Fadini 2017 and Nur Aisyah 2020

Seven studies reporting the results of TC changes relative to baseline included 2696 patients. The pooled analysis revealed a significant difference in TC changes relative to baseline between Dapagliflozin and Placebo [MD = 3.12; 95% CI (0.64, 5.60); *P* = 0.01], with low heterogeneity (I^2^ = 25%). Compared with alternative studies, the research by Fadini revealed in 2017, and the research by Nur Aisyah revealed in 2020 manifested prominent heterogeneity. After deleting the above two studies, heterogeneity was low (I^2^ = 0%). Not only that, the comprehensive effects revealed that the TC changes relative to baseline further became an extremely significant difference in both groups [MD = 3.34; 95% CI (1.88, 4.79); *P* < 0.00001]. In the research by Fadini and Nur Aisyah, the dapagliflozin group had a significantly lower BMI and body weight at baseline compared to the placebo group. In contrast, other studies did not have such significant differences in baseline BMI and body weight. The publication bias of TC changes relative to baseline was assessed in the Egger's test, and no publication bias was detected (Fig. 3).

##### Meta-analysis of the LDL-C changes relative to baseline

**Fig. 4:**
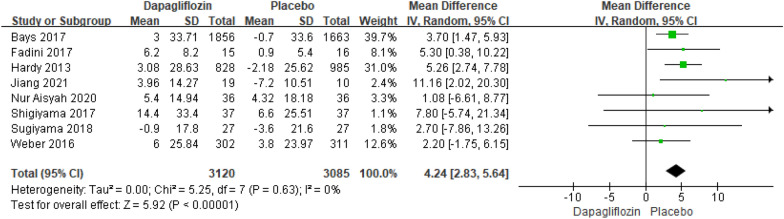
Impact of dapagliflozin on LDL-C changes relative to baseline

Eight studies reporting the results of LDL-C changes relative to baseline included 6205 patients. The pooled analysis resulted in a highly significant difference in LDL-C changes relative to baseline between dapagliflozin and placebo [MD = 4.24; 95% CI (2.83, 5.64); *P* < 0.00001], with low heterogeneity (I^2^ = 0%). The publication bias of LDL-C changes relative to baseline was assessed in the Egger's test, which showed no publication bias (Fig. 4).

##### Meta-analysis of the HDL-C changes relative to baseline

**Fig. 5:**
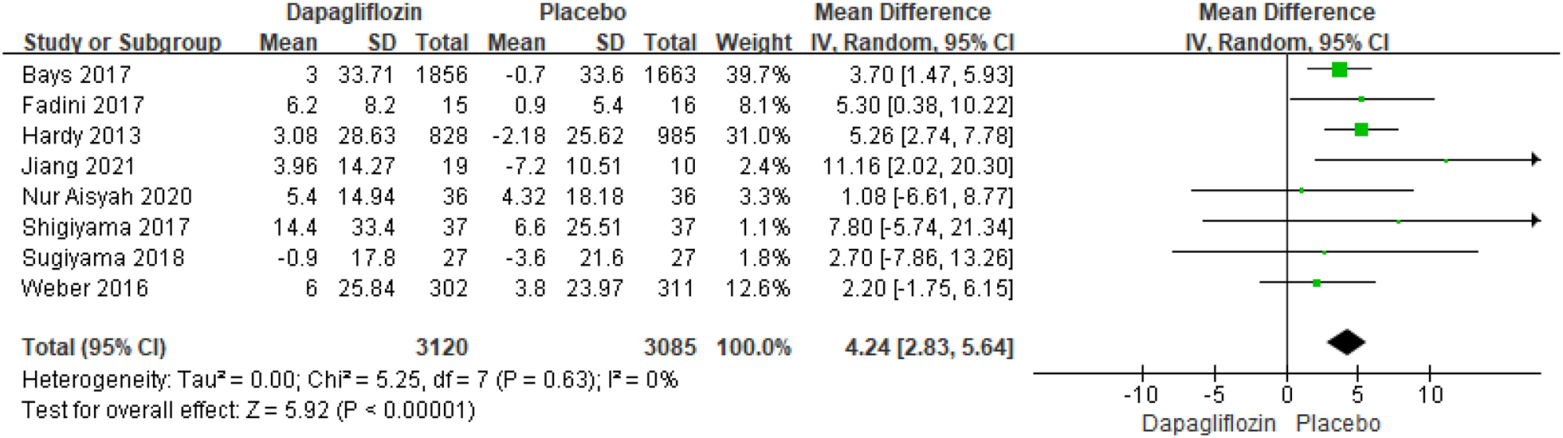
Impact of dapagliflozin on HDL-C changes relative to baseline after deleting Fadini 2017 and Nur Aisyah 2020

Eight studies reporting the results of HDL-C changes relative to baseline included 6241 patients. There was no significant distinction in HDL-C changes relative to baseline [MD = 0.40; 95% CI (− 1.02, 1.82); *P* = 0.58], with high heterogeneity (I^2^ = 77%). Compared with alternative studies, the research by Fadini revealed in 2017, and the research by Nur Aisyah revealed in 2020 manifested prominent heterogeneity. After deleting the above two studies, heterogeneity was low (I^2^ = 0%). Not only that, the comprehensive effects revealed that the HDL-C changes relative to baseline change to an extremely significant difference in both groups [MD = 1.39; 95% CI (0.77, 2.01); *P* < 0.0001]. In the research by Fadini and Nur Aisyah, the dapagliflozin group had a significantly lower BMI and body weight at baseline compared to the placebo group, while other studies did not have such significant differences in baseline BMI and body weight. The publication bias of HDL-C changes relative to baseline was assessed in the Egger's test, no publication bias was detected (Fig. 5).

##### Meta-analysis of the TG changes relative to baseline

**Fig. 6:**
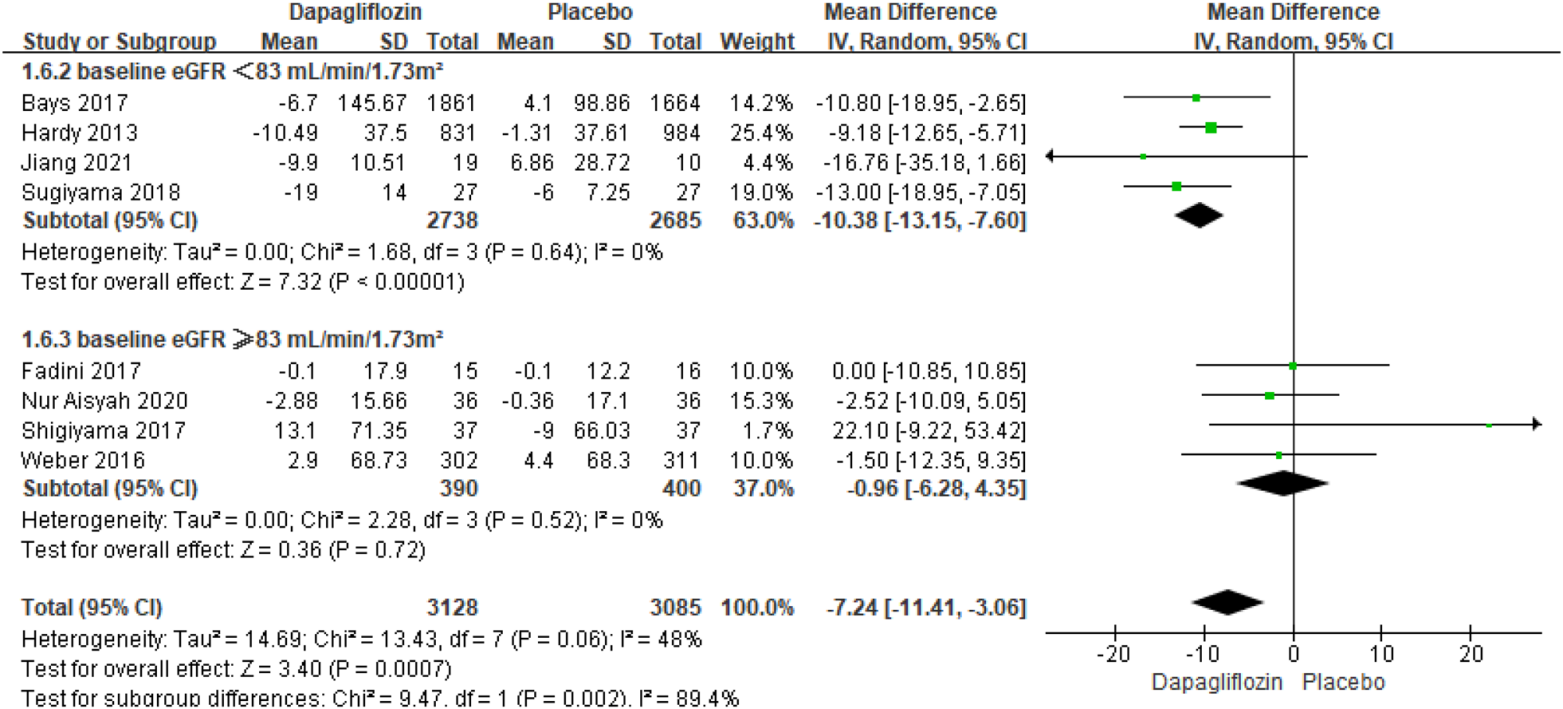
Impact of dapagliflozin on TG changes relative to baseline

Eight studies reporting the results of TG changes relative to baseline included 6213 patients. The pooled analysis showed that there was an extremely significant difference in TG changes relative to baseline between dapagliflozin and placebo [MD = − 7.24; 95% CI (− 11.41, − 3.06); *P* = 0.0007], but the heterogeneity is high (I^2^ = 48%). Throughout the subgroup analysis, we found that in the subgroups whose patient's baseline eGFR < 83 ml/min/1.73m^2^, dapagliflozin considerably decreased the TG levels (*P* < 0.00001), with lower heterogeneity (I^2^ = 0%). However, in the subgroups whose patient's baseline eGFR ≥ 83 ml/min/1.73m^2^, there was no difference in dapagliflozin and placebo (*P* = 0.72). The Egger's test assessed the publication bias of TG changes relative to baseline, which showed no publication bias (Fig. 6).

##### Meta-analysis of the adiponectin changes relative to baseline

**Fig. 7:**
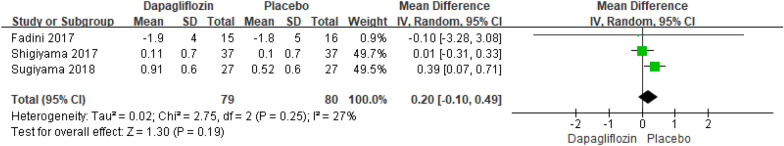
Impact of dapagliflozin on adiponectin changes relative to baseline

Three studies reporting the results of adiponectin changes relative to baseline included 159 patients. The pooled analysis showed that there was no difference in adiponectin changes relative to baseline between dapagliflozin and placebo [MD = 0.20; 95% CI (− 0.10, 0.49); *P* = 0.19], with low heterogeneity (I^2^ = 27%). No publication bias was detected according to Egger's test (Fig. 7).

#### Hemodynamic parameter and endothelial function

##### Meta-analysis of the SBP changes relative to baseline

**Fig. 8:**
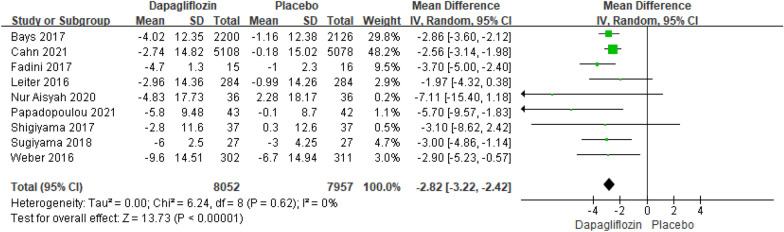
Impact of dapagliflozin on SBP changes relative to baseline

Nine studies reporting the results of SBP declines relative to baseline included 16,009 patients. The pooled analysis showed that there was an extremely significant difference in SBP declines relative to baseline between dapagliflozin and placebo [MD = − 2.82; 95% CI (− 3.22, − 2.42); *P* < 0.00001], with low heterogeneity (I^2^ = 0%). The publication bias of SBP declines relative to baseline was assessed in the Egger's test, which showed no publication bias (Fig. 8).

##### Meta-analysis of the DBP changes relative to baseline

**Fig. 9:**
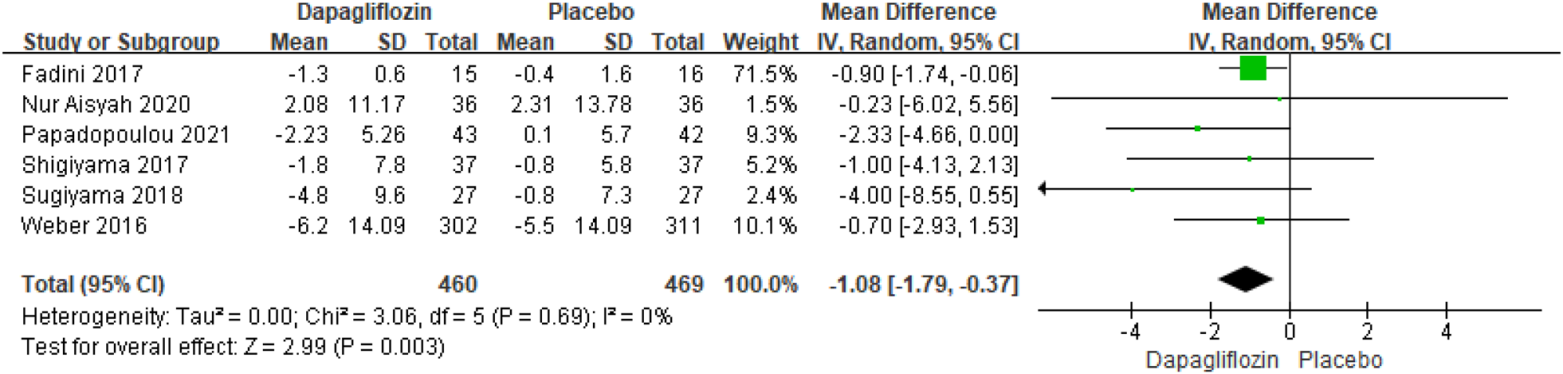
Impact of dapagliflozin on DBP changes relative to baseline

Six studies reporting the results of DBP declines relative to baseline included 929 patients. The pooled analysis showed that there was an extremely significant difference in DBP declines relative to baseline between dapagliflozin and placebo [MD = − 1.08; 95% CI (− 1.79, − 0.37); *P* = 0.003], with low heterogeneity (I^2^ = 0%). The publication bias of DBP declines relative to baseline was assessed in the Egger's test, which showed no publication bias (Fig. 9).

##### Meta-analysis of the ΔFMD changes relative to baseline

**Fig. 10:**
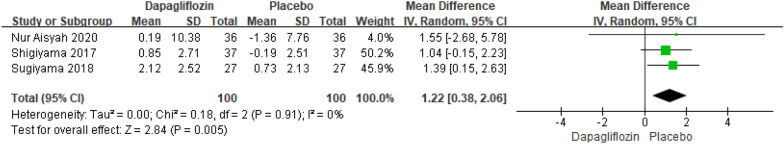
Impact of dapagliflozin on ΔFMD changes relative to baseline

Three studies reporting the results of ΔFMD changes relative to baseline included 200 patients. The pooled analysis resulted in an extremely significant difference in ΔFMD changes relative to baseline between dapagliflozin and placebo [MD = 1.22; 95% CI (0.38, 2.06); *P* = 0.005], with low heterogeneity (I^2^ = 0%). The Egger's test assessed the publication bias of ΔFMD changes relative to baseline, which showed no publication bias (Fig. 10).

#### Glycemic control

##### Meta-analysis of the HbA1c changes relative to baseline

Nine studies reporting the results of HbA1c changes relative to baseline included 15,930 patients. The pooled analysis resulted in a significant difference in HbA1c changes relative to baseline between dapagliflozin and placebo [MD = − 0.47; 95% CI (− 0.70, − 0.25); *P* < 0.0001], but the heterogeneity of recurrence rate is extremely high (I^2^ = 97%). Throughout the subgroup analysis and sensitivity analysis, we tend to find that in both subgroups whose patient's mean age < 60 years and patient's mean age ≥ 60 years, dapagliflozin respectively considerably decreased the HbA1c levels (*P* < 0.00001), with lower heterogeneity (I^2^ = 0%).

**Fig. 11:**
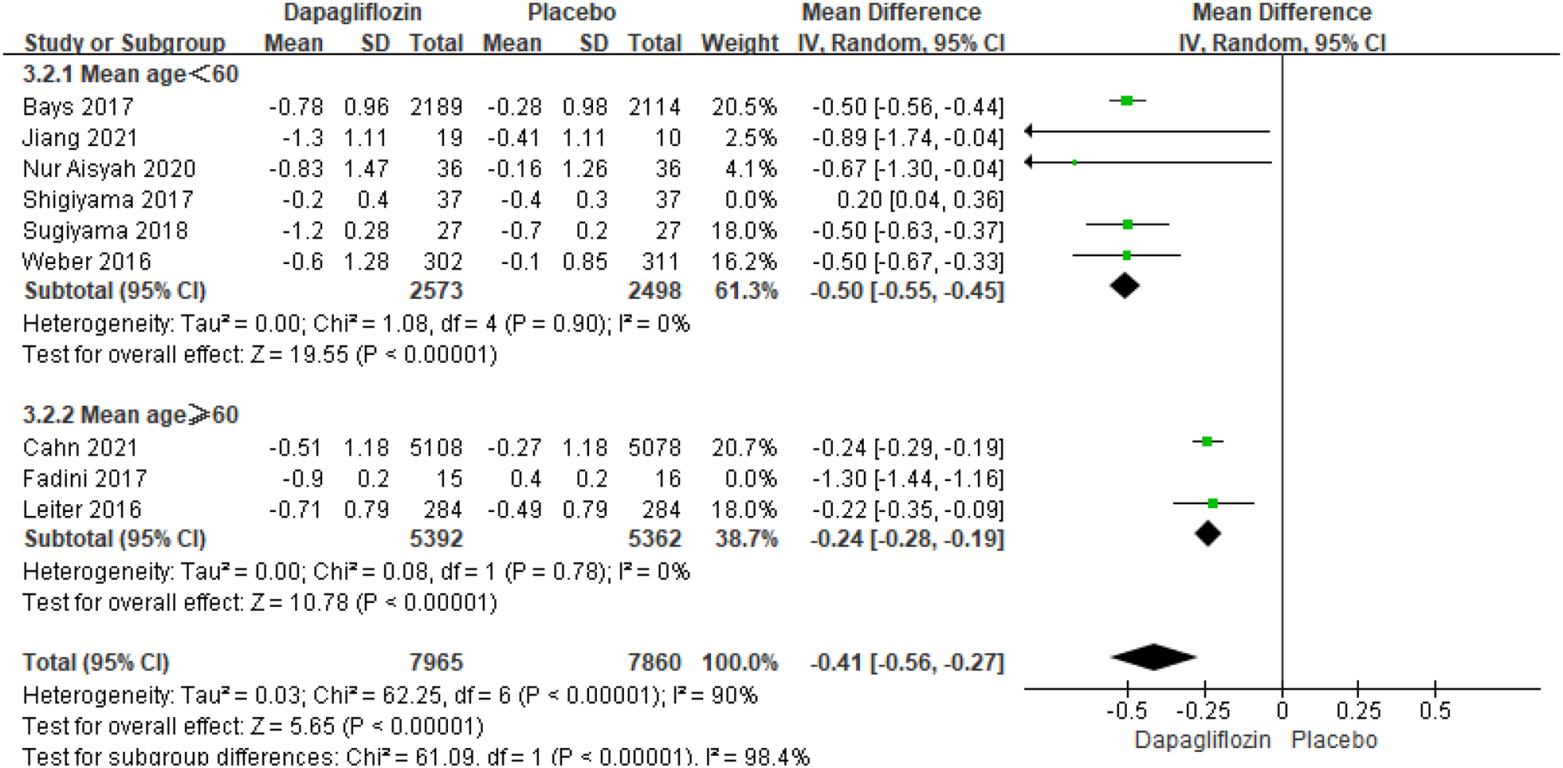
Impact of dapagliflozin on HbA1c changes relative to baseline after deleting Fadini 2017 and Shigiyama 2017

Compared with alternative studies, the research by Shigiyama revealed in 2017 and research by Fadini revealed in 2017, respectively manifested prominent heterogeneity in the subgroup of mean age < 60 and subgroup of mean age ≥ 60. In the research by Shigiyama, the mean duration of T2DM of the patients was six years, while other studies were more than 7.5 years. On the other side, the baseline BMI and bodyweight of the patients in the dapagliflozin group were prominently lower than the patient in the group of placebos in the research by Fadini, while other studies did not have such significant differences in baseline BMI and body weight. The publication bias of HbA1c changes relative to baseline was assessed in the Egger's test, which showed no publication bias (Fig. 11).

#### Metabolic parameter

##### Meta-analysis of the bodyweight changes relative to baseline

**Fig. 12:**
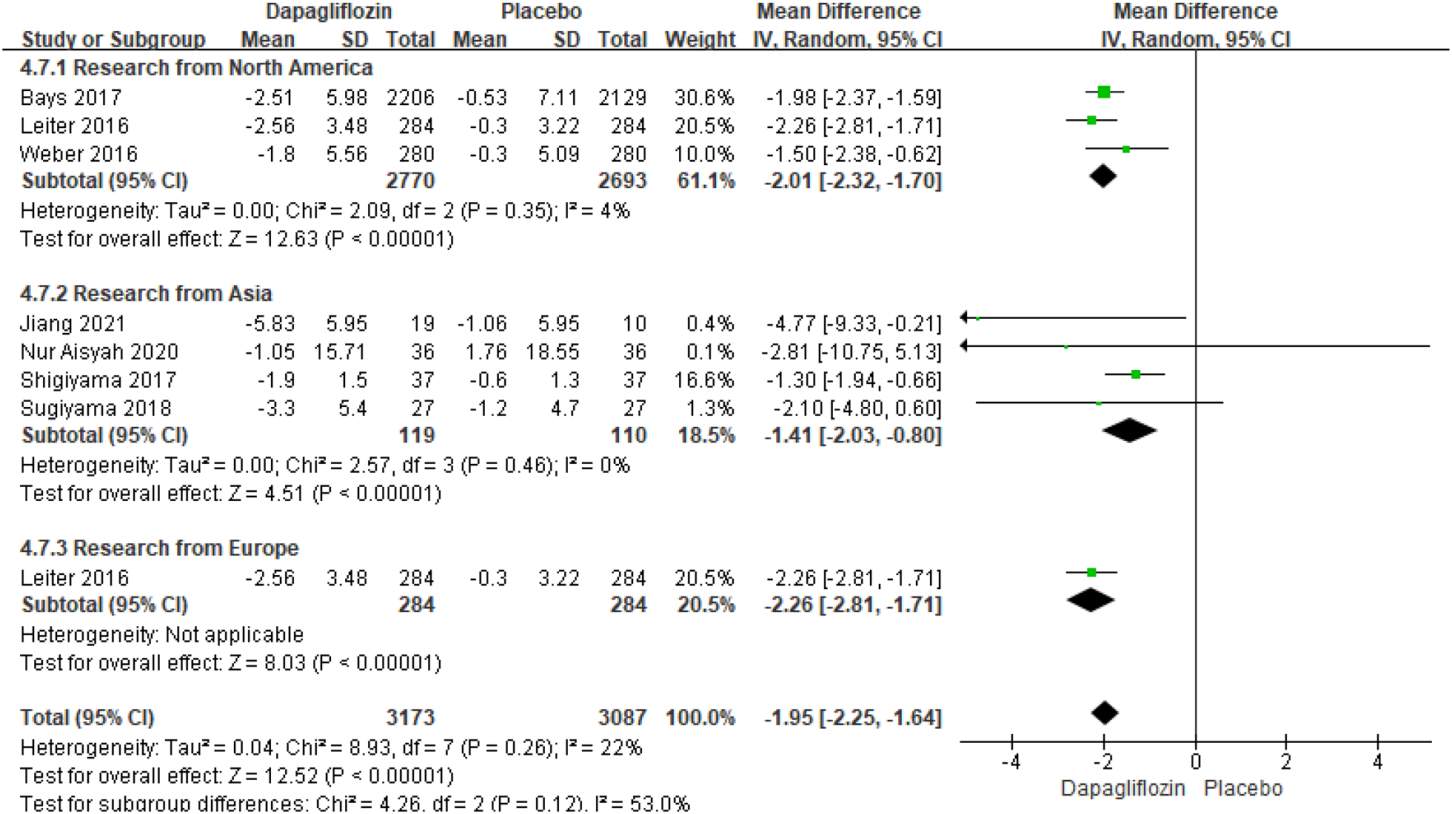
Impact of dapagliflozin on body weight changes relative to baseline

Eight studies reporting the results of body weight changes relative to baseline included 6260 patients. The pooled analysis showed that there was an extremely significant difference in body weight changes relative to baseline between dapagliflozin and placebo [MD = − 1.95; 95% CI (− 2.25, − 1.64); *P* < 0.00001], but there is an inevitable heterogeneity of recurrence rate(I^2^ = 22%). Throughout the subgroup analysis, we tend to find that dapagliflozin respectively considerably decreased the body weight changes relative to baseline in the subgroups whose research from both North American (*P* < 0.00001, I^2^ = 4%) and Asia (*P* < 0.00001, I^2^ = 0%). Nevertheless, other studies from Europe and Oceania cannot be determined. The publication bias of body weight changes relative to baseline was assessed in the Egger's test, which showed no publication bias (Fig. 12).

##### Meta-analysis of the BMI changes relative to baseline

**Fig. 13:**
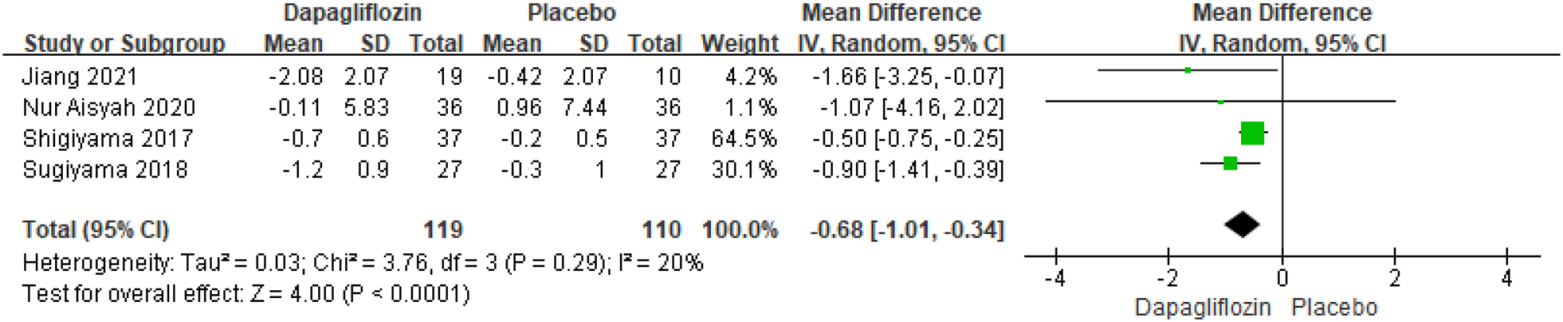
Impact of dapagliflozin on BMI changes relative to baseline

Four studies reporting the results of BMI changes relative to baseline included 229 patients. The pooled analysis showed that there was a pronounced significant difference in BMI changes relative to baseline between dapagliflozin and placebo [MD = − 0.68; 95% CI (− 1.01, − 0.34); *P* < 0.0001], with low heterogeneity (I^2^ = 20%). The publication bias of BMI changes relative to baseline was assessed in the Egger's test, which showed no publication bias (Fig. 13).

##### Meta-analysis of the waist circumference changes relative to baseline

**Fig. 14:**
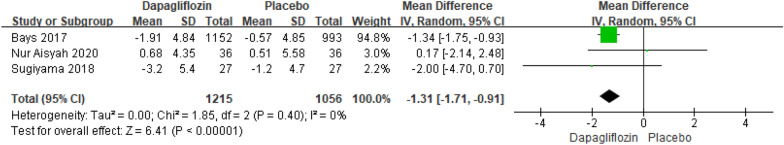
Impact of dapagliflozin on waist circumference changes relative to baseline

Three studies reporting the results of waist circumference changes relative to baseline included 2271 patients. The pooled analysis showed that there was a pronounced significant difference in waist circumference changes relative to baseline between dapagliflozin and placebo [MD = − 1.31; 95% CI (− 1.71, − 0.91); *P* < 0.00001], with low heterogeneity (I^2^ = 0%). The publication bias of waist circumference changes relative to baseline was assessed in the Egger's test, which showed no publication bias (Fig. 14).

##### Meta-analysis of the C-peptide immunoreactivity changes relative to baseline

**Fig. 15:**
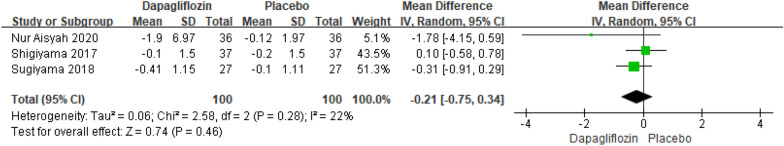
Impact of dapagliflozin on C-peptide immunoreactivity relative to baseline

Three studies reporting the results of C-peptide immunoreactivity changes relative to baseline included 200 patients. The pooled analysis showed that there was no difference in C-peptide immunoreactivity changes relative to baseline between dapagliflozin and placebo [MD = − 0.21; 95% CI (− 0.75, 0.34); *P* = 0.46], with low heterogeneity (I^2^ = 22%). Egger's test showed no publication bias (Fig. 15).

##### Meta-analysis of the eGFR changes relative to baseline

**Fig. 16:**
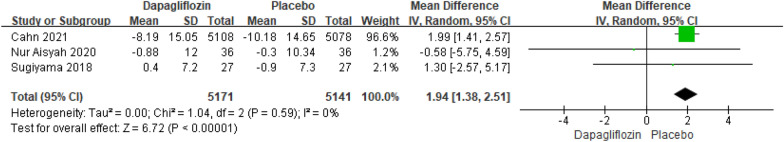
Impact of dapagliflozin on eGFR relative to baseline

Three studies reporting the results of eGFR changes relative to baseline included 10,312 patients. The pooled analysis showed that there was a pronounced significance in eGFR changes relative to baseline between dapagliflozin and placebo [MD = 1.94; 95% CI (1.38, 2.51); *P* < 0.00001], with middle heterogeneity (I^2^ = 0%). Egger's test showed no publication bias (Fig. 16).

## Discussion

Type 2 diabetes mellitus, a chronic disease, can lead to a higher risk of cardiovascular diseases and even death. In addition, the risk of coronary heart disease increases in T2DM patients by 11% for each 1% increment in HbA1c greater than 6.5% [23]. Additional hazard factors for coronary heart disease in patients with T2DM have increased consistencies of LDL-C and decreased consistencies of HDL-C, all of which are high-risk factors of atherosclerosis [24]. Based on the increased risk of ASCVD with T2DM, the treatment for not only decreasing HbA1c levels but also controlling or preventing the progression of atherosclerosis is vital to patients with T2DM. At present, dapagliflozin has been proved to benefit HbA1c and cardiovascular outcomes for T2D patients [13], whereas HDL-C and LDL-C targets of patients are not met with dapagliflozin [14].

At present, an increasing number of studies are trying to determine whether dapagliflozin has a significant effect on the occurrence and development of atherosclerosis in patients with T2DM, but there is no consensus. In addition, the former meta-analyses, relying on only a few previous studies and a minimal number of research indicators, have not been able to draw sufficient conclusions simultaneously. Therefore, we conducted a meta-analysis to evaluate the effectiveness of dapagliflozin in the occurrence and development of atherosclerosis in patients with T2DM.

Our study included 12 RCTs of 18,758 patients, seven of which were high quality and five low qualities.

Concerning lipid parameters, as we all know, high plasma levels of HDL-C and low plasma levels of TG and LDL-C are protective factors for atherosclerosis [25]. The oxidative modification of lipids, especially the fatty acyl residues in phospholipids, can lead to the formation of free radicals and cell damage. Lipid peroxidation products in ox-LDL can induce the inflammatory phenotype of arterial wall cells, which leads to endothelial dysfunction and apoptotic cell death, which is a critical step in the occurrence and development of atherosclerotic lesions [26]. HDL-C can prevent the oxidative damage of LDL-C caused by free radicals through various effects, such as antioxidation, anti-thrombosis, anti-infection, anti-apoptosis, and anti-inflammation [27]. On the other hand, unlike LDL-C, triglycerides-rich lipoproteins can be directly ingested by macrophages, which can be transformed into marker cells of atherosclerotic plaques, that is, macrophage foam cells rich in indigestible cholesterol droplets, which is a major cause of arterial plaque formation [28]. In this connection, our results showed that compared with placebo, dapagliflozin could better increase HDL-C concentrations and decrease TG plasma levels. Among them, the decrease in plasma levels of TG was only evident in subgroups of baseline eGFR < 83 ml/min/1.73m^2^, while the subgroup of baseline eGFR ≥ 83 ml/min/1.73m^2^ did not. The different effects of eGFR on the effect of dapagliflozin changing TG's plasma levels may be the following reason. First of all, the chronic low eGFR often causes delayed catabolism, which reduces the activity of peripheral lipoprotein lipase and hepatic triglyceride lipase, and it will cause the increase of TG [29]. However, it is fortunate that dapagliflozin could significantly protect the eGFR, according to our study, which eventually controlled the plasma levels of TG. Judging from the above results, dapagliflozin might reduce the incidence and progression of atherosclerosis in diabetic patients. Moreover, the effect is more significant in diabetic patients with kidney disease.

The majority view LDL-C as an essential hazard factor for atherosclerosis. In the early stage of the lesion, LDL-C accumulates in the intima, which leads to the formation of reactive oxygen species (the Fenton reaction) under the catalysis of metal ions, and then accelerates the formation of atherosclerotic plaque [30]. Nevertheless, our pooled analysis found that LDL-C changes relative to the baseline of the dapagliflozin group were significantly higher than placebo, which suggested that dapagliflozin harms atherosclerosis. But even then, LDL-C can be divided into large buoyant particles (lb) and small dense particles (sd) according to size and density. A large number of clinical evidence shows that sd-LDL-C particles are more likely to cause atherosclerosis than lb-LDL-C particles, and the advantage of sd-LDL-C increases the risk of coronary artery disease by three times [31] because sd-LDL-C forms a suitable substrate for oxidized LDL-C on the arterial wall [32]. In the present study, the increase of LDL-C level caused by dapagliflozin, whether the increase of sd-LDL-C is dominant or the increase of lb-LDL-C is dominant, or the level of both is similar, the factors are not apparent. Therefore, the risk factors of increased LDL-C for atherosclerotic progression need to be further clarified. Coincidentally, the change of TC is also in line with the above the law; nonetheless, it is worth noting that TC cannot be used as an indicator of atherosclerosis risk alone because it reflects the sum of all lipoprotein in blood and the accurate level of HDL-C, LDL-C, TG and so forth are difficult to assess.

Moreover, it is worth noting that adiponectin was positively correlated with HDL-C and negatively correlated with LDL-C and TG concentrations [33]. Zhang et al. found that the level of circulating adiponectin was negatively correlated with the risk of coronary heart disease [34]. Unfortunately, our results did not show that dapagliflozin has an influence on adiponectin. On the whole, the protection of the incidence and progression of atherosclerosis by dapagliflozin is challenging to judge from the changes in lipid parameters, and a series of more in-depth studies are needed.

In terms of hemodynamic parameters and endothelial function, our pooled analysis found that the SBP and DBP declines relative to the baseline of the dapagliflozin group were significantly higher than the placebo. As we all know, high systolic blood pressure and diastolic blood pressure is a hazard factor for atherosclerosis [35], on account of the changes in connective permeability associated and endothelial tissue metabolism with atherosclerosis are increased by hypertension [36]. Consequently, the effective control of SBP and DBP by dapagliflozin could reduce the incidence and progression of atherosclerosis in diabetic patients. It happens that there is a similar case in Δflow-mediated vasodilatation. In this regard, Matsuzawa et al. [37] reported that an increase of 1% in Δflow-mediated vasodilatation is associated with a 12% reduction in adjusted relative risk of future atherosclerosis. In our study, dapagliflozin could significantly increase Δflow-mediated vasodilatation, which suggests dapagliflozin may protect incidence and progression of atherosclerosis in diabetic patients. In addition, Leng W et al. [38] showed that dapagliflozin not only alleviated the formation of atherosclerosis in diabetic animals, but also effectively increased plaque stability. This effect may be related to its inhibition of ROS-NLRP3-caspase-1 pathway activity in atheromatous plaque macrophages of diabetic mice, which further provides useful evidence for its application in diabetic patients.

With regard to glycemic control and metabolic parameter, our pooled analysis found that in both subgroups whose patients' mean age < 60 years and patient's mean age ≥ 60 years, and dapagliflozin enormously decreased HbA1c levels relative to baseline in contrast with placebo. Furthermore, our study has also illustrated that dapagliflozin had a tremendous advantage in controlling all three of BMI, waist circumference, and eGFR relative to baseline. What is more, dapagliflozin immensely decreased body weight relative to baseline in the subgroups whose research was from both North America and Asia. In a related matter, overweight and obesity are associated with low-grade inflammation, leading to plaque growth and complications, namely rupture and thrombosis [39]. It has been observed that this marker is associated with increased fibrinogen transcapillary escape rate and increased von Willebrand factor levels in diabetic patients with reduced eGFR, suggesting that decreased eGFR may reflect general endothelial dysfunction and systemic vascular injury [40]. So, the above indications are all suggesting that dapagliflozin reduced the relative risk of future atherosclerosis. Of note, in relation to placebo, dapagliflozin was no significant distinction in C-peptide immunoreactivity changes relative to baseline.

We get the following results when the present study is compared with other existing meta-analyses. First and foremost, Cai et al. [41] carried out a meta-analysis of 55 placebo-controlled trials suggesting that changes in body weight relative to baseline were associated with a dose of dapagliflozin treatment. In our pooled analysis, the bodyweight changes relative to baseline have an extreme difference between dapagliflozin and placebo. However, we only included a small number of studies of 10 mg and 5 mg of dapagliflozin; therefore, the association of body weight changes and dapagliflozin treatment dosage was unable to evaluate. One more point is that our pooled analysis of lipid metabolism has partly consisted of the meta-analysis of Musso et al. [42], which included seven studies with dapagliflozin; dapagliflozin improved HDL-C while not in triglyceride, total, and LDL-cholesterol. What makes the difference is, we found that the subgroups of baseline eGFR < 83 ml/min/1.73m^2^ had a significant decrease on TG level while the subgroup of baseline eGFR ≥ 83 ml/min/1.73m^2^ did not. Fortunately, in the study of Handelsman et al. [43] it concluded that the decrease of TG is favorable to improve atherosclerotic cardiovascular disease (ASCVD) outcomes. Therefore, considering the results of our analysis, it is reasonable to suggest that dapagliflozin reduces the risk of atherosclerosis in T2DM patients. However, in Musso's study [42], no similar results were obtained for TG levels in the dapagliflozin group. Meanwhile, Jabbour et al. [44] performed a meta-analysis including 13 placebo-controlled trials and suggested that no meaningful changes were found in lipid variables. Last but not least, Sonesson et al. [45] conducted a meta-analysis including 21 studies and found that with or without a history of CVD, dapagliflozin can reduce the risk of MACE, which supported our experimental results on a certain level.

When considering these results, several limitations should be aware of. First and foremost, in the results of dapagliflozin's evaluation of lipid parameters, the increase of HDL-C and the decrease of TG, as well as the increase of LDL-C and TC, have no way to evaluate which is superior to the progression of atherosclerosis. In addition, the factors that cannot be manipulated constantly may influence, such as different ethnicity, ages at the initiation of intervention, duration of intervention, formulation of dapagliflozin and placebo, and dietary and exercise guidelines. There is one more point that the inclusive literature may be potentially biased during the review process. Last but not least, we included a small number of articles and only incorporated the published studies, which may impact the results. Further research is needed on this aspect.

In recent years, oral hypoglycemic agents can alter the natural history of diabetes by reducing the risk of cardiovascular events, heart failure hospitalizations, and renal disease. SGLT-2 inhibitors are increasingly accepted as novel glucose-lowering agents and have become a key new therapeutic agent for clinicians managing patients with diabetes at high risk for comorbid CVD or CV. There are still many large clinical studies ongoing with SGLT-2 inhibitors, including those evaluating effects on heart failure: EMPEROR-Reduced, EMPEROR-Preserved, Dapa-HF, DELIVER; those evaluating effects on diabetic nephropathy: Dapa-CKD, EMPA-KIDNEY; and those evaluating effects on hypertension: PREHYPD. It is believed that the systemic vascular protective effects of dapagliflozin-led SGLT-2 inhibitors will be increasingly appreciated through various clinical studies. In terms of the underlying mechanism, it is still unclear whether it is a direct or systemic effect of dapagliflozin and its specific pathways and related mRNAs and proteins. Furthermore, the specific mechanisms related to the changes of dapagliflozin expression in the vasculature in disease states are not yet understood and clarified. In addition, factors such as gender, age, diabetes, glycemic control, dapagliflozin compounds, and the presence of comorbidities may alter the effect of dapagliflozin on atherosclerosis, and the impact of these factors and their specific mechanisms remain unclear and need to be further explored in subsequent studies. Therefore, more studies are required to investigate the application of dapagliflozin in the vasculature and in atheromatous plaques for better clinical application.

## Conclusions

Our pooled analysis suggested that dapagliflozin has a terrifically better influence over HDL-C, ΔFMD, and eGFR, and it concurrently had a tremendous advantage in controlling TG, SBP, DBP, HbA1c, BMI, body weight, and waist circumference, but it also harms increasing TC and LDL-C. Furthermore, this study found that the effect of dapagliflozin on decreasing plasma levels of TG was only evident in subgroups of baseline eGFR < 83 ml/min/1.73m^2^ while the subgroup of baseline eGFR ≥ 83 ml/min/1.73m^2^ did not. Finally, the above results summarize that dapagliflozin could be a therapeutic option for the progression of atherosclerosis in patients with T2DM.

## Data Availability

The datasets used and analyzed during the current study are available from the corresponding author on reasonable request.

## Abbreviations

ASCVD: Atherosclerotic cardiovascular disease
T2DM: Type 2 diabetes mellitus
SGLT2i: Sodium-dependent glucose transporters two inhibitors
TC: Total cholesterol
LDL-C: Low-density lipoprotein-cholesterol
HDL-C: High-density lipoprotein-cholesterol
TG: Triglycerides
SBP: Systolic blood pressure
DBP: Diastolic blood pressure
△FMD: Flow-mediated vasodilatation
HbA1c: Hemoglobin A1c
FPG: Fasting plasma glucose
BMI: Body mass index
CPR: C-peptide immunoreactivity
eGFR: Estimated glomerular filtration rate
UACR: Urine albumin creatine ratio
RCT: Randomized controlled trial
NRCT: Non-randomized controlled trial
NCT No.: National clinical trial number
SD: Standard deviation
95% CI: 95% Confidential interval
OR: Odds ratio
MD: Mean difference
